# Serological biomarker for assessing human exposure to *Aedes* mosquito bites during a randomized vector control intervention trial in northeastern Thailand

**DOI:** 10.1371/journal.pntd.0009440

**Published:** 2021-05-27

**Authors:** Benedicte Fustec, Thipruethai Phanitchat, Sirinart Aromseree, Chamsai Pientong, Kesorn Thaewnongiew, Tipaya Ekalaksananan, Dominique Cerqueira, Anne Poinsignon, Eric Elguero, Michael J. Bangs, Neal Alexander, Hans J. Overgaard, Vincent Corbel

**Affiliations:** 1 Univ Montpellier, Montpellier, France; 2 Khon Kaen University, Khon Kaen, Thailand; 3 MIVEGEC, Univ Montpellier, IRD, CNRS, Montpellier, France; 4 Department of Medical Entomology, Faculty of Tropical Medicine, Mahidol University, Bangkok; 5 HPV & EBV and Carcinogenesis Research Group, Khon Kaen University, Khon Kaen, Thailand; 6 Office of Diseases Prevention and Control, Region 7, Khon Kaen, Thailand; 7 Public Health & Malaria Control, International SOS, Mimika, Papua, Indonesia; 8 Department of Entomology, Faculty of Agriculture, Kasetsart University, Bangkok, Thailand; 9 London School of Hygiene and Tropical Medicine, London, United Kingdom; 10 Norwegian University of Life Sciences, Ås, Norway; University of Oxford, UNITED KINGDOM

## Abstract

**Background:**

*Aedes* mosquitoes are vectors for several major arboviruses of public health concern including dengue viruses. The relationships between *Aedes* infestation and disease transmission are complex wherein the epidemiological dynamics can be difficult to discern because of a lack of robust and sensitive indicators for predicting transmission risk. This study investigates the use of anti-*Aedes* saliva antibodies as a serological biomarker for *Aedes* mosquito bites to assess small scale variations in adult *Aedes* density and dengue virus (DENV) transmission risk in northeastern Thailand. Individual characteristics, behaviors/occupation and socio-demographics, climatic and epidemiological risk factors associated with human-mosquito exposure are also addressed.

**Methods:**

The study was conducted within a randomized clustered control trial in Roi Et and Khon Kaen provinces over a consecutive 19 months period. Thirty-six (36) clusters were selected, each of ten houses. Serological and entomological surveys were conducted in all houses every four months and monthly in three sentinel households per cluster between September 2017 and April 2019 for blood spot collections and recording concurrent immature and adult *Aedes* indices. Additionally, the human exposure to *Aedes* mosquito bites (i.e., Mosquito Exposure Index or MEI) was estimated by ELISA measuring levels of human antibody response to the specific Nterm-34 kDa salivary antigen. The relationships between the MEI, vector infestation indices (adult and immature stages) and vector DENV infection were evaluated using a two-level (house and individual levels) mixed model analysis with one-month lag autoregressive correlation.

**Results:**

There was a strong positive relationship between the MEI and adult *Aedes* (indoor and outdoor) density. Individuals from households with a medium mosquito density (mean difference: 0.091, p<0.001) and households with a high mosquito density (mean difference: 0.131, p<0.001) had higher MEI’s compared to individuals from households without *Aedes*. On a similar trend, individuals from households with a low, medium or high indoor *Aedes* densities (mean difference: 0.021, p<0.007, 0.053, p<0.0001 and 0.037, p<0.0001 for low, medium and high levels of infestation, respectively) had higher MEI than individuals from houses without indoor *Aedes*. The MEI was driven by individual characteristics, such as gender, age and occupation/behaviors, and varied according to climatic, seasonal factors and vector control intervention (p<0.05). Nevertheless, the study did not demonstrate a clear correlation between MEI and the presence of DENV-infected *Aedes*.

**Conclusion:**

This study represents an important step toward the validation of the specific IgG response to the *Aedes* salivary peptide Nterm-34kDa as a proxy measure for *Aedes* infestation levels and human-mosquito exposure risk in a dengue endemic setting. The use of the IgG response to the Nterm-34 kDa peptide as a viable diagnostic tool for estimating dengue transmission requires further investigations and validation in other geographical and transmission settings.

## Author summary

*Aedes* mosquitoes and the viruses they transmit are major public health concerns for over half of the global human population. However, the quantitative relationships between virus transmission and vector mosquito infestation remain unclear despite numerous indicators used to estimate transmission risk and predict dengue outbreaks. The aim of this study is to investigate the use of a salivary biomarker to assess the small-scale variation in human exposure to *Aedes* bites and the risk of dengue infection in the context of a vector control intervention in northeastern Thailand. A cohort of 539 persons visited every four months, including 161 individuals visited monthly, were recruited for routine serological and concurrent household entomological surveys during 19 consecutive months follow-up. Antibody response to *Aedes* bites was measured by enzyme-linked immunosorbent assays to assess the mosquito exposure index (MEI) and association with the *Aedes* adult and immature abundance as well as the presence of dengue virus (DENV) in adult mosquitoes (transmission risk). Additionally, the individual (cohort), climatic, and vector control intervention risk factors associated with MEI are explored. This study demonstrates that the MEI was strongly related to household adult *Aedes* density, particularly indoors resting mosquitoes. Additionally, the MEI was influenced by individual characteristics (i.e., person age, gender, staying indoors), and varied according to seasons and intervention. Nonetheless, no clear relationship between MEI and dengue transmission risk (i.e., vector infection) was detected. This study demonstrated the potential usefulness of the MEI to assess heterogeneity in adult *Aedes* infestation indices that could assist public health authorities to rapidly identify mosquito “hot spots” and the timeliness of effective vector control interventions.

## Introduction

*Aedes aegypti* (L) and *Aedes albopictus* (Skuse) are vectors of important human viral pathogens including dengue, yellow, chikungunya and Zika. In Southeast Asia, dengue fever is widespread and accounts for around 70% of the total clinical dengue cases reported globally [1,2]. Since the first report of dengue infection in Thailand in 1949 [3], dengue incidence has dramatically increased in line with expanding urbanization. With all four virus serotypes and both major mosquito vectors present in the country around 20,000 cases are reported yearly [4]. Despite an affordable, universal primary health coverage system and an organized, nationwide dengue prevention program, the burden of dengue in Thailand is estimated to cost the equivalent of at $290 million (USD) each year [5].

In northeastern Thailand, dengue fever represents major public health concerns with thousands of clinical cases each year [6]. To prevent secondary transmission in communities, when a dengue case is detected, insecticide treatment using adult space spray is mandated within 24 hours in attempt to rapidly eliminate virus-infected vectors, surrounding its home setting [7]. In parallel, basic entomological surveillance is carried regularly by one of the 22 regional Offices of Diseases Prevention and Control (ODPCs) to monitor *Aedes* vector infestations [7]. In Thailand standard entomological indices are used to estimate transmission risk that guide the choice of vector control interventions [8]. While, some studies have shown positive associations between various entomological indices and disease transmission risk [9,10], other investigations have demonstrated only weak relationships [11–13]. Most of the entomological indices used to monitor dengue vector infestations are based on measuring the presence of immature mosquito life stages [14]. However, immature stages typically present large mortality rates during development from egg to adult stage [15], thus larval indices do not provide an accurate or concurrent temporal-spatial information on the ‘productivity’ of containers regards actual *Aedes* adult production output [16]. Conversely, pupal indices have been proposed to assess vector infestation with higher accuracy [17,18] as pupae generally present very low mortality up to adult emergence and thus more relevant to estimate container productivity [19] and adult densities in a location [16]. Operationally, pupae collections remain difficult to implement on a routine basis because they are time-consuming (generally all pupae must be collected and counted) that requires additional entomological staff.

Adult mosquito collections have been used to estimate the risk of virus transmission [19,20], but they have also their limitations. Unlike malaria vector monitoring, human land-catching cannot be performed to collect *Aedes* mosquitoes due to the inherent ethical constraints and disease risks, as there is no preventive treatment nor effective vaccines for most of *Aedes*-transmitted diseases/pathogens (except yellow fever virus). Moreover, *Aedes* adults are most active during the day time, when most people are awake and can take some forms of protection against bites. As a consequence, *Aedes* females are often interrupted in the course of seeking a blood meal and can often feed on multiple hosts per gonotrophic cycle [21–23]. Other methods to sample adult *Aedes* include various versions of passive and active trapping devices (e.g., gravitraps, sticky traps, mechanical battery-operated aspirators, and mosquito electrocuting trap) [24], each presenting differing levels of efficiency [25]. However, they do not measure the inter-individual heterogeneity of exposure influenced by human attraction exerted on mosquitoes and individual host behaviors (e.g., use of personal protections). Nevertheless, these capture methods are used as a proxy to estimate *Aedes* adult density in a specific area but they are not representative of actual level of contact (biting) exposure between human and vector [26]. This information is yet crucial to identify host population subsets at higher risk of exposure to dengue vector bites and to better estimate virus transmission risk.

An alternative to direct entomological indices for estimating the human exposure to mosquitoes is the measure of a host’s antibody (Ab) response to mosquito saliva antigens [27–29]. During blood feeding process, mosquito saliva is initially injected into human skin to facilitate the blood intake and also acts as a vehicle for transmitting pathogens to the host [30]. Many salivary proteins are immunogenic and elicit an immune response including the production of specific antibodies (Ab) that can be detected by simple analytic tools and spectrophotometry [31–33]. Firstly developed for *Anopheles*, the vectors of malaria, so-called biomarkers of exposure based on anti-saliva Ab response have been used successfully to identify “hot spots” of vector presence and malaria transmission [34–36] along the Thailand-Myanmar border [34,37]. As far as *Aedes* genus is concerned, several other studies have shown that IgG response to salivary gland extracts from different *Aedes* species, such as *Ae*. *aegypti*, *Ae*. *polynesiensis*, *Ae*. *caspius* are reliable indicators of human-*Aedes* exposure in South-America [38,39], Pacific Islands [40], Africa [41] and Europe [31]. An *Ae*. *aegypti*-specific salivary peptide (Nterm-34 kDa) has been identified and the human IgG response to the Nterm-34 kDa antigen has shown good correlation with adult *Ae*. *aegypti* infestation indices in Benin [42] and Laos [43]. More recently, the Nterm-34 kDa salivary peptide successfully investigated the spatial heterogeneity of *Aedes* exposure in several urban districts of Senegal [44]. However, most of these *Aedes* serological studies estimated vector infestation through “indirect” (relative) indicators such as immature ‘*Stegomyia*’ (*Aedes*) indices and climatic factors, thus were unlikely to represent more accurate adult infestation that which is directly associated with virus transmission potential. Robust evidence of the relationships between the intensity of human immune response to a specific salivary biomarker, *Aedes* adult abundance, and dengue infective bite risk is needed to assess whether small scale variations in dengue transmission can be detected using this immunological tool. This is particularly relevant for measuring the impact of vector control interventions where entomological indices may lack the spatio-temporal accuracy and sensitivity to demonstrate control effectiveness [16,45,46].

The primary objective of this study was to assess the relationship between the intensity of the human IgG response to the Nterm-34kDa *Aedes* salivary peptide and selected entomological indicators of vector infestation and dengue infection risk in northeastern Thailand. This study took place within the context of a randomized controlled trial implemented over a consecutive 19-month period to evaluate the efficacy of an insect growth regulator tool for dengue transmission prevention [47,48]. Additionally, risk factors associated with human-vector contact in terms of individual human characteristics and behavior, local vector control practices, and the prevailing seasonal and climatic factors were addressed. To our knowledge, this is the first longitudinal study conducted to assess dengue transmission risk using a serological *Aedes* salivary biomarker. Hopefully, these findings will assist national authorities to improve the accuracy of dengue surveillance activities and contribute to strengthening the monitoring and evaluation of vector control programs in Thailand and elsewhere.

## Materials and methods

### Ethics statement

This trial was registered (ISRCTN, ISRCTN73606171) and approved by the Khon Kaen University Ethics Committee (KKUEC Record No. 4.4.01: 29/2017, Reference No. HE601221, 1 September 2017), the London School of Hygiene and Tropical Medicine Ethical Committee, UK (LSHTM Ethics Ref: 14275, 16 August 2017), and the Regional Committee for Medical and Health Research Ethics, Section B, South East Norway (REK Ethics ref: 2017/1826b, 03 March 2018). Each participant was informed about the intent of the study and asked to participate on a voluntary basis. In each household, the head of the house signed a consent form to allow periodic entomological inspection inside and outside their residence. Additionally, signed informed consent (or assent, if under 16 years old) were required each time blood samples were taken.

### Study sites

**Fig 1:**
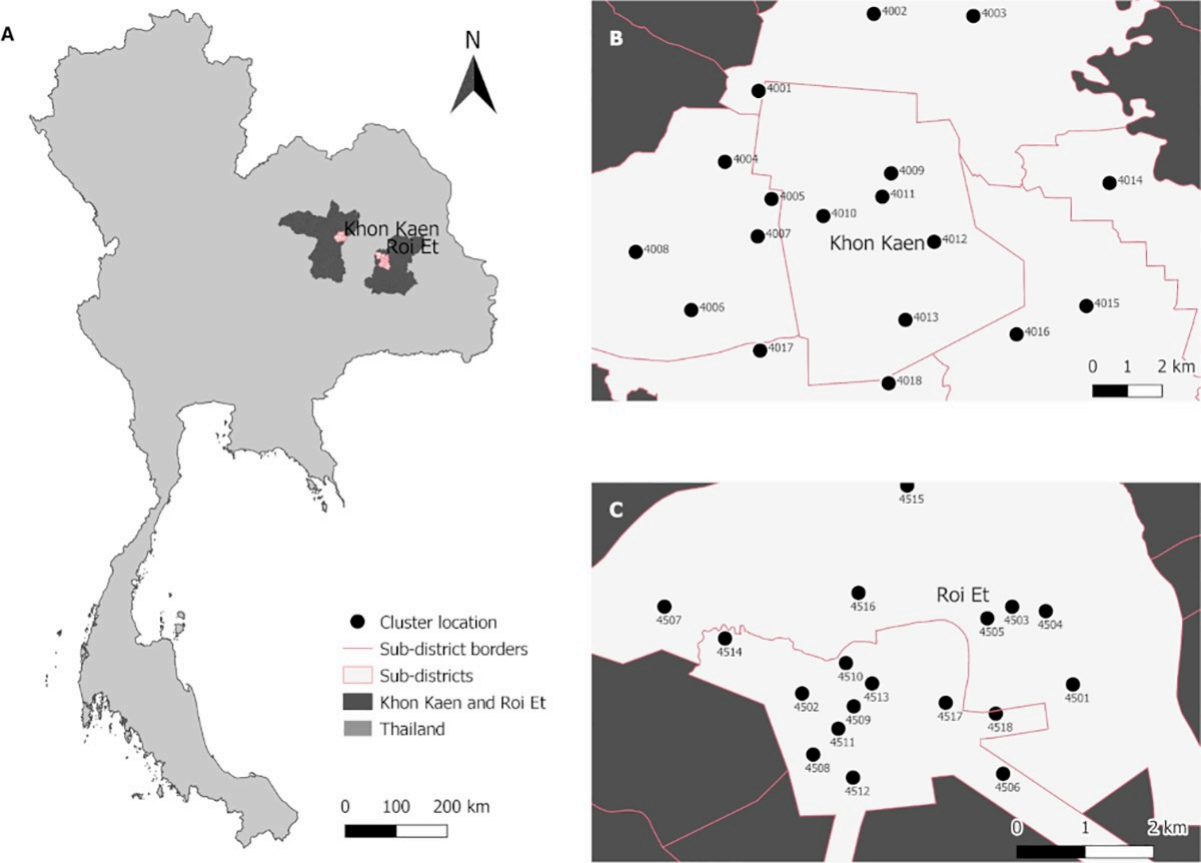
Map of study sites. (A) represents Thailand and the provinces of Roi Et and Khon Kaen. (B) shows the location of the 18 clusters numbered from 4001 to 4018 in the city of Khon Kaen (KK Mueang District). (C) shows the location of the 18 clusters numbered from 4501 to 4518 in the city of Roi Et (RE Mueang District). Map of study sites was built using QGis 3.10 software and shapefiles were obtained from the Humanitarian Data Exchange project [49] under the Creative Commons Attribution International 4.0 license (CC BY 4.0).

The study was conducted in six sub-districts in the city of Khon Kaen (KK), Khon Kaen Province, (N16.440236, E102.828272) and in two sub-districts within the city of Roi Et (RE), Roi Et Province, (N16.055637, E103.652417), in northeastern Thailand (Fig 1). In each city, 18 clusters of 10 households each were randomly selected for a total of 360 households under 19 months of follow-up.

### Study design and settings

**Fig 2:**
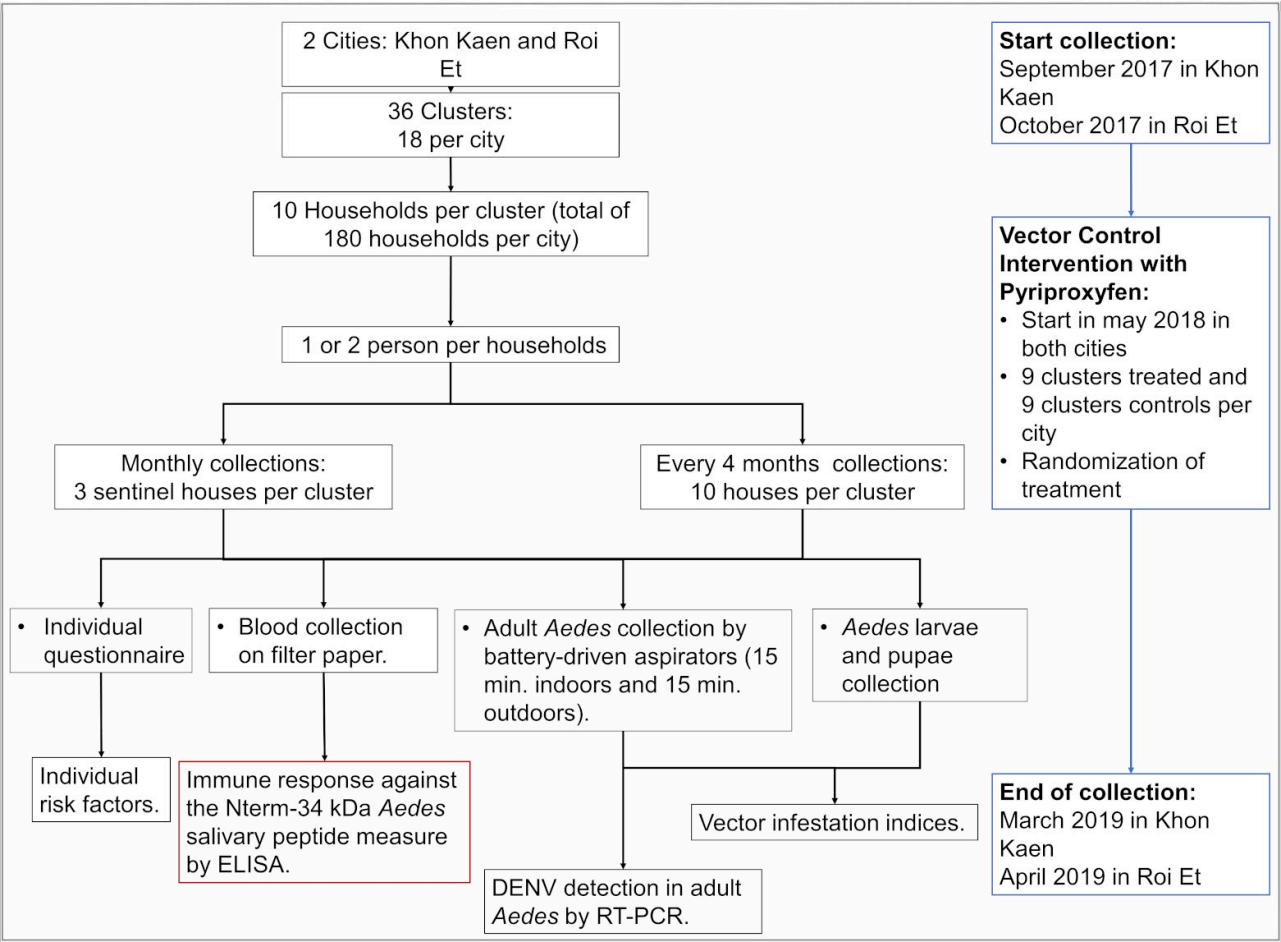
Study design flow chart. RT-PCR: Reverse Transcriptase Polymerase Chain Reaction.

This study was conducted within the framework of a randomized control intervention trial to evaluate the efficacy of pyriproxyfen application (0.5% granule formulation) for dengue vector control [47,48]. The study was performed in Khon Kaen between September 2017 and March 2019 and between October 2017 and April 2019 in Roi Et (Fig 2). All households were visited every four months (except one time in RE between February 2018 and May 2018) to collect indoor and outdoor container-breeding *Aedes* (both *Ae*. *aegypti* and *Ae*. *albopictus)* larvae, pupae, adult resting mosquitoes, and blood samples from study volunteers living in randomly selected households. In addition, three sentinel houses per cluster were visited monthly for blood and entomological collections described previously. Following the initial 10 months of baseline surveillance, the vector control intervention was distributed randomly in half of the study clusters, in June 2018. The household selection in the cities and the randomization of the intervention are described elsewhere [47,48]. The vector control intervention was the distribution of pyriproxyfen (0.5% granule formulation) into water-holding containers up to 0.01 mg/L active ingredient applied every four months in the treated clusters [47,48].

### Individual volunteer characteristics

In each participating household, at least one volunteer inhabitant was recruited in the study. When possible, we tried to recruit inhabitants spending most of their time at home. To ensure adequate representativeness of the entire target population, we recruited one adult and one child per house when feasible. In addition, a pecuniary retribution (50THB) for blood sampling was given to each participant. During each household visit, assigned trained Village Health Volunteers (VHV) interviewed and collected blood of each participating house member. Interview questions were relative to the general characteristics of the participant (i.e., age, gender), occupation(s) during the weekdays and weekends (e.g., at home; at work away from home; at school/college/university; at farm; others), in addition to normal activity and resting habits (i.e., primarily indoor, outdoor or equally indoor and outdoor). The travelling history within the previous 14 days and within the last three months was recorded.

### Blood sample collections

Blood samples (2 blood spots per participant, 10mm diameter each, approximately 150μl) were collected at the fingertips of the inhabitants recruited in the study using sterile lancets [50] and spotted on filter paper Protein Saver cards (Whatman, Maidstone, UK), air-dried, individually placed in plastic sealable bags and stored at room temperature at the Office of Disease Prevention and Control 7 (ODPC7) until delivery to Khon Kaen University (KKU) and stored at 4°C.

### Entomological collections

At each household visit, the VHVs recorded the number of inhabitants in the household at the time of the survey. Houses were inspected for adult and immature *Aedes* both indoor and outside immediately surrounding the house. The total number of containers was recorded together with the number of wet containers at each household. A maximum of 20 larvae (preferably late stage instar) and all pupae were collected per infested container and stored in absolute ethanol at the ODCP7. Immatures and adults were identified to species-level using morphological keys [51,52], and sex was determined for adults. *Aedes* adult collections were performed using hand-held mechanical battery-powered aspirators [53] conducted 15 min each both indoors and outdoors. Adults were stored individually in labelled 1.5mL microcentrifuge tubes at -20°C and the house number and the location of collection (i.e., indoor/outdoor) was recorded.

Entomological data were used to construct several indices as described in Supporting information S1 Table and *Aedes* indices described hereafter include both *Ae*. *aegypti* and *Ae*. *albopictus*. At the cluster level, the Container Index (CI_c_) was calculated as the proportion of *Aedes* immature-positive containers per total wet containers inspected in all visited households at the time of survey. The cluster-wide Breteau Index (BI_c_) and the House Index (HI_c_) were calculated as the proportion of *Aedes* positive containers per 100 houses and the proportion of positive households visited, respectively. The cluster-level pupal indices, Pupae per House Index (PHI_c_) and the Pupae per Person Index (PPI_c_), represented the total number of pupae collected per household and per inhabitants in each visited household, respectively. The *Aedes* Index (AI_c_) and the *Aedes* indoor Index (AI_in_c_) at the cluster level represented the total number of female *Aedes* collected per inspected houses and the total number of female *Aedes* collected exclusively indoors, respectively.

### Detection of dengue virus in adult mosquitoes

The presence of dengue virus (DENV) in *Aedes* females was investigated in all captured adult mosquitoes, by pooling up to 10 individual abdomens of female *Aedes* together for RNA extraction and DENV detection by reverse transcriptase real-time polymerase chain reaction (RT-qPCR) [54]. For positive pools, the head and thorax of the corresponding individual mosquitoes were processed individually for DENV serotype detection according to Lanciotti *et al* protocol and adapted by our team to be run on RT-qPCR [54]. The proportion of DENV infected *Aedes* was calculated as the number of DENV infected individual *Aedes* divided by the number of tested *Aedes* females per house (AI DENV+) and per cluster (AI_c_ DENV+), respectively.

### Climatic data

The Meteorological Department of Thailand provided climatic data routinely recorded from the meteorological stations located at the airport of each city [55]. Daily measures were used to derive the minimum and maximun air temperatures (°C), the percent relative humidity, and the rainfall (mm) between January 2016 to January 2020. For analysis, the mean maximum and minimum temperatures, mean percent relative humidity, and cumulative rainfall the previous two weeks before entomological collections were used to account for an estimated time-lag effect on vector population biology and transmission epidemiology.

### Mosquito Exposure Index (MEI)

The specific human IgG response to the Aedes Nterm-34kDa salivary peptide (Genepep, Saint Jean de Védas, France) was measured by an enzyme-linked immunosorbent assay (ELISA) as described previously [48,56]. This secreted salivary peptide was selected because it exhibits high antigenic properties and it is specific to *Aedes* genus, therefore allowing to specifically measure the immune response to *Aedes* bites alone [42]. Briefly, for each individual sampled, dried blood spots were cut using a one cm diameter hole punch and eluted in 400μl of Phosphate Buffer Saline (PBS) for 24h at 4°C. The resulting eluates were stored at -20°C until further processing. 96-well Maxisorp micro-assay plates (Nunc, Roskilde, Denmark) were coated with the salivary peptide diluted in PBS (20μg/mL) for 180 minutes at 37°C. Following washing and blocking steps, the blood eluates were diluted at 1:160 in PBS containing 1% of Tween20 (1%-PBST) and incubated overnight at 4°C. ELISA plates were incubated with goat anti-human biotin-conjugated IgG (Invitrogen, Thermo Scientific, USA) diluted at 1:6000 in 1%- PBST for 90 min at 37°C, followed by streptavidin horseradish peroxidase (GE Healthcare, Amersham Place, UK) diluted at 1:4000 in 1%-PBST for one hour at 37°C. The colorimetric reaction was performed using ABTS buffer (2,2’-azino-bis (3-ethylbenzthiazoline 6-sulfonic acid) di-ammonium) + 0.003% H_2_O_2_ and absorbance (optical density, OD) was measured after 120 min at 405nm with a Sunrise spectrophotometer (Tecan, Switzerland).

All samples were assayed in duplicate and in a blank well (no antigen) to measure individual background and antibody response (ΔOD) expressed as:

$$\Delta OD=\mathrm{mean}\;({\mathrm{OD}}_{\mathrm{Ag}+})‐{\mathrm{OD}}_{\mathrm{Ag}‐}$$ (1)

where "OD_Ag+_ " represents the OD value in the well with the salivary antigen and "OD_Ag-_ " the OD value in the well without the antigen.

To quantify the non-specific immune reactions and calculate the immune threshold, anti- Nterm-34kDa IgG response was assayed in individuals (n = 16) with no known exposure history to *Ae*. *aegypti* bites [57] (e.g., dry blood spots collected in northern France from January to March 2016 to 2018, and in Western Australia in October 2016). The specific immune threshold (TR) was defined as follows at0.556.


$$TR=mean\;(\Delta {\mathrm{OD}}_{\mathrm{unexposed}\;\mathrm{individuals}})+3\;SD_{\mathrm{unexposed}\;\mathrm{individuals}}$$ (2)

We also defined the Mosquito Exposure Index (MEI) for each participant as

MEI=ΔOD−TR. (3)

The MEI represents the level of specific and individual IgG response to the *Aedes* salivary peptide. Individuals with a ΔOD value above the TR, thus with a positive MEI, were classified as “immune responders” (i.e., exposed to *Aedes*). Individuals with a ΔOD value equal or below the TR, and therefore with a null or negative MEI value, were categorized as “non-responders” (i.e., non-exposed to *Aedes*). Individuals with negative or null MEI were considered equally having a null MEI as the background immune response cannot be addressed.

### Analysis

#### Covariates

The human study population was stratified into five age groups: 5–19, 20–39, 40–59, 60–69, and ≥70 years of age. Individual’s characteristics were analyzed as categorical variables to estimate their influence on the MEI. Overall travel history of each subject was used as a binary variable. At the village level, adult *Aedes* indices recorded one-month before blood collection, and immatures *Aedes* indices recorded at the time of survey were used. Additionally, the pyriproxyfen intervention was used as a binary covariate. At the province level, the mean daily maximum and minimum air temperatures, mean percent relative humidity, and the weekly cumulative rainfall two weeks before collections were treated as covariates. The estimated 2-week time-lag takes into account potential influence on vector population biology and transmission epidemiology. Three general climatic seasons are defined according to the Thai Meteorological Department [55] with 15-February to 14-May as the hot season, 15-May to 14-October as the rainy (wet) season, and 15 October to 14-February as the cool season.

#### Statistical approach

Data analysis was conducted using R software version 3.5.1 (R Core Team, Vienna, Austria) and MASS, Rcmdr, nlme4, and lmerTest packages [58–60]. Figures were generated on R using ggplot2 and ggpubr packages [61,62]. Maps were built using QGIS software (version 3.10) and shape files were obtained from the Humanitarian Data Exchange Project [49]. As the MEI represents the specific exposition to *Ae*. *aegypti*, non-responder individuals were considered with a null MEI, thus the MEI was considered as a positive continuous variable (i.e., MEI ≥0). The relation between MEI and entomological indices was explored using a multivariate 2-level mixed model (house, individual) with a one-month lag autoregressive correlation, assuming the antibody response persisted at detectable levels between two and six weeks [33,63]. The (1) *Aedes* adult index (2) *Aedes* adult indoor index, and (3) proportion of DENV-infected *Aedes* at the cluster level were examined in three separate analyzes. A fourth analysis was conducted with the proportion of DENV-infected *Aedes* at the household level to assess the heterogeneity of dengue transmission risk between and within study clusters. To avoid the assumption of linear relationships between antibody response to *Aedes* bites and entomological indices, risk factors were categorized into categorical variables to represent the different levels of intensity. Due to the over dispersion of mosquito numbers over time, immature stages and adult entomological indices at the cluster level were categorized into four classes, the null value of the index, and then following the terciles. The presence of DENV-infected *Aedes* was used as a binary variable (0 or >0) due to the low number of sampled DENV-infected *Aedes*. All analyzes were performed on individuals with complete data, while individuals with missing data in covariates of interest were removed. Univariable analysis using a mixed model was conducted with each covariate to identify adjustment factors related to immune response to Nterm-34 kDa for all models. Multivariable mixed models were performed with all covariates with a *p*-value set at < 0.2. Subsequently, models were adjusted by backward selection and removing non-significant variables at *p*-value < 0.05.

## Results

### Population characteristics

The studied population, 602 individuals (318 in KK and 284 in RE), were followed-up every four months up to 19 months for an average of 3.5 visits per person (Table 1) producing a total of 3,919 collected dried blood spot samples. Among the 602 individuals recruited, a sub- sample of 92 and 71 individuals in KK and RE, respectively, were followed-up each month in sentinel sites with an average of 14.7 visits per person. The majority of the cohort was female (65.3% and 69.0% in KK and RE, respectively). The median age of the cohort was 64 and 61 years in KK and RE, respectively. The majority of the study cohort stayed most of the time at home during the weekdays and weekends (Table 2); although, in KK, about 30% of the cohort, mostly those of younger age, indicated spending some time in schools during the weekends. In KK, the vast majority of the individuals spent their weekdays indoors while in RE, about one fifth spent their weekdays both indoors and outdoors (near the location where they spend their time). Nevertheless, the behavioral trend was quite similar between KK and RE regarding daytime activities (e.g., indoor vs. outdoor locations). Most individuals were primarily sedentary with >95% declaring no travel in the previous 3 months before blood collections. At the time of the study, there was no evidence of incident (new) dengue infection; therefore, results presented herein is performed using entomological and immunological data only.

**Table 1:** Population description and immunological status to Nterm-34 kDa salivary peptide.

		Khon Kaen		Roi Et	
Population size, n individuals (no. dried blood spots)		318	(2003)	284	(1916)
Age in years, median (range of all participants)		64	(5–90)	61	(7–92)
Female proportion, % (no. females/total)		65.3	(1307/2003)	69.0	(1319/1916)
Dengue cases %, (no. cases/total)		0.00	(0/2003)	0.00	(0/1916)
Proportion of immune responder during the whole study, %, (no. responding/total)	All ages	57.3	(1148/2003)	60.0	(1150/1916)
	Age 5–19	46.7	(14/30)	53.8	(21/39)
	Age 20–39	48.9	(66/135)	64.7	(119/184)
	Age 40–59	58.9	(367/623)	60.2	(415/689)
	Age 60–69	58.2	(322/553)	54.0	(299/554)
	Age 70+	57.3	(379/662)	65.8	(296/450)

**Table 2:** Individual participant characteristics, behavior and occupation. (NA: Not available).

		Khon Kaen		Roi Et	
		No. individuals = 318		No. individuals = 284	
**Occupation weekdays, %, (no. answers/total)**	Home	90.8	(1818/2003)	93.8	(1797/1916)
	Work away from home	7.19	(144/2003)	0.47	(9/1916)
	School/college/university	0.70	(14/2003)	0.68	(13/1916)
	Farm	1.10	(22/2003)	0.05	(1/1916)
	Other	0.10	(2/2003)	0.00	(0/1916)
	NA	0.15	(3/2003)	5.01	(96/1916)
**Occupation weekends, %, (no. answers/total)**	Home	69.3	(1388/2003)	94.2	(1805/1916)
	Work away from home	1.34	(27/2003)	0.05	(1/1916)
	School/college/university	29.3	(587/2003)	7.31	(14/1916)
	Farm	0.00	(0/2003)	0.05	(1/1916)
	Other	0.05	(1/2003)	0.00	(0/1916)
	NA	0.00	(0/2003)	4.96	(95/1916)
**Location spent weekdays, %, (no. answers/total)**	Indoor	94.6	(1895/2003)	67.4	(1291/1916)
	Outdoor	3.10	(64/2003)	0.68	(13/1916)
	Indoor and outdoor	2.00	(40/2003)	19.9	(382/1916)
	NA	0.20	(4/2003)	12.0	(230/1916)
**Location spent weekends, %, (no. answers/total)**	Indoor	46.0	(922/2003)	55.7	(1068/1916)
	Outdoor	0.50	(10/2003)	0.05	(1/1916)
	Indoor and outdoor	26.0	(521/2003)	18.5	(355/1916)
	NA	25.0	(550/2003)	25.7	(492/1916)
**Travel in the last 14 days, %, (no. answers/total)**	No	96.5	(1932/2003)	94.4	(1808/1916)
	Yes	3.54	(71/2003)	0.68	(13/1916)
	NA	0.00	(0/2003)	4.96	(95/1916)
**Travel in the last 3 months, %, (no. answers/total)**	No	95.3	(1909/2003)	91.6	(1756/1916)
	Yes	4.70	(94/2003)	3.390	(65/1916)
	NA	0.00	(0/2003)	4.96	(95/1916)
**Travel overall during study, % (no. answers/total)**	No	92.3	(1848/2003)	91.4	(1752/1916)
	Yes	7.70	(155/2003)	3.60	(69/1916)
	NA	0.00	(0/2003)	4.96	(95/1916)

### Entomological collections and indices

Overall, 2,235 resting adults female *Aedes* were captured, of which the vast majority, 1,772 (79.3%) were collected indoors (Table 3). *Aedes aegypti* was the overwhelmingly predominant species identified (99.7%) compared to *Aedes albopictus* with only seven females *Ae*. *albopictus* collected. In Khon Kaen, 1,397 females *Aedes* (including *Ae*. *aegypti* and *Ae*. *albopictus*) were collected during a combined 1,446 house visits, the large majority (77%) captured indoors (Table 3). Moreover, DENV infection was detected among 16 females *Aedes* in KK. In Roi Et, 838 females *Aedes* were sampled from 1,441 collections, of which 696 (83%) were collected indoors. Moreover, DENV was detected among 14 females *Aedes* in RE. Additionally, 992 *Aedes* pupae (544 in KK and 448 in RE) were collected in the two cities. As with adult mosquitoes, *Ae*. *aegypti* pupae represented the vast majority (95.7%) of collections, therefore, all *Aedes* indices were estimated using *Ae*. *aegypti* and *Ae*. *albopictus* altogether. At the cluster level, the standard larval indices (CI_c_, HI, BI) indicated significantly higher *Aedes* infestation in Khon Kaen compared to Roi Et with an average of 16.4% and 4.11% *Aedes* positive containers, respectively (S2 Table). Similarly, the adult *Aedes* indices (AI_c_ and AI_in_c_) were higher in KK clusters than in RE, with an average of 3.7 and 1.0 *Aedes* in KK and 0.79 and 0.68 *Aedes* in RE, respectively. Only the DENV-infected adult *Aedes* index (AI_c_ DENV+) was higher in RE clusters than in KK with an average of 0.007 and 0.005 proportion of DENV positive *Aedes* in RE and KK, respectively. The pupal indices were, however, slightly higher in RE than in KK with 0.84 and 0.63 PHI_c_ and 0.26 and 0.19 PPI_c_, respectively.

**Table 3:** Entomological collection data and indices at household and cluster level.

	**Khon Kaen**			**Roi Et**		
	Houses	Visits	Total	Houses	Visits	Total
*Aedes* female collected	179	1446	1397	179	1441	838
*Aedes* female collected indoors	179	1446	1076	179	1441	696
*Aedes* pupae collected	179	1446	544	179	1441	448
**Entomological indices**						
	Mean	Std dev	Range	Mean	Std dev	Range
**Household level**						
Adult Index DENV+	0.005	0.049	[0–1]	0.005	0.057	[0–1]
**Cluster level**						
Container Index_c_ (CI_c_) (%)	16.4	14.8	[0–100]	4.11	9.17	[0–66.7]
House Index_c_ (HI_c_)(%)	45.5	33.8	[0–100]	12.9	21.3	[0–100]
Breteau Index_c_ (BI_c_)	60.4	55.4	[0–300]	14.2	25.0	[0–137.5]
Pupae per House Index_c_ (PHI_c_)	0.63	1.40	[0–10.7]	0.84	1.99	[0–10.7]
Pupae per Person Index_c_ (PPI_c_)	0.19	0.45	[0–3.3]	0.26	0.72	[0–5.7]
Adult Index_c_ (AI_c_)	3.71	2.42	[1–15]	0.79	0.84	[0–6]
Adult Index_indoor_c_ (AI_in_c_)	1.00	0.87	[0–5]	0.68	0.71	[0–4]
Adult Index_c_ DENV+	0.005	0.035	[0–0.33]	0.007	0.057	[0–0.67]

### Spatial and seasonal variation in mosquito exposure and vector density

During the study, 3,919 individual dried blood samples were collected and processed, including 2,003 and 1,916 in KK and RE, respectively. The seroprevalence rates for IgG reactivity were 57.3% and 60% in KK and RE, respectively, indicating that most individuals exhibited a specific response to the Nterm-34kDa *Ae*. *aegypti* salivary peptide (Table 1). The proportion of immune responders between combined RE and KK clusters was not statistically significant (χ^2^ p = 0.08) (S2 Table).

**Fig 3:**
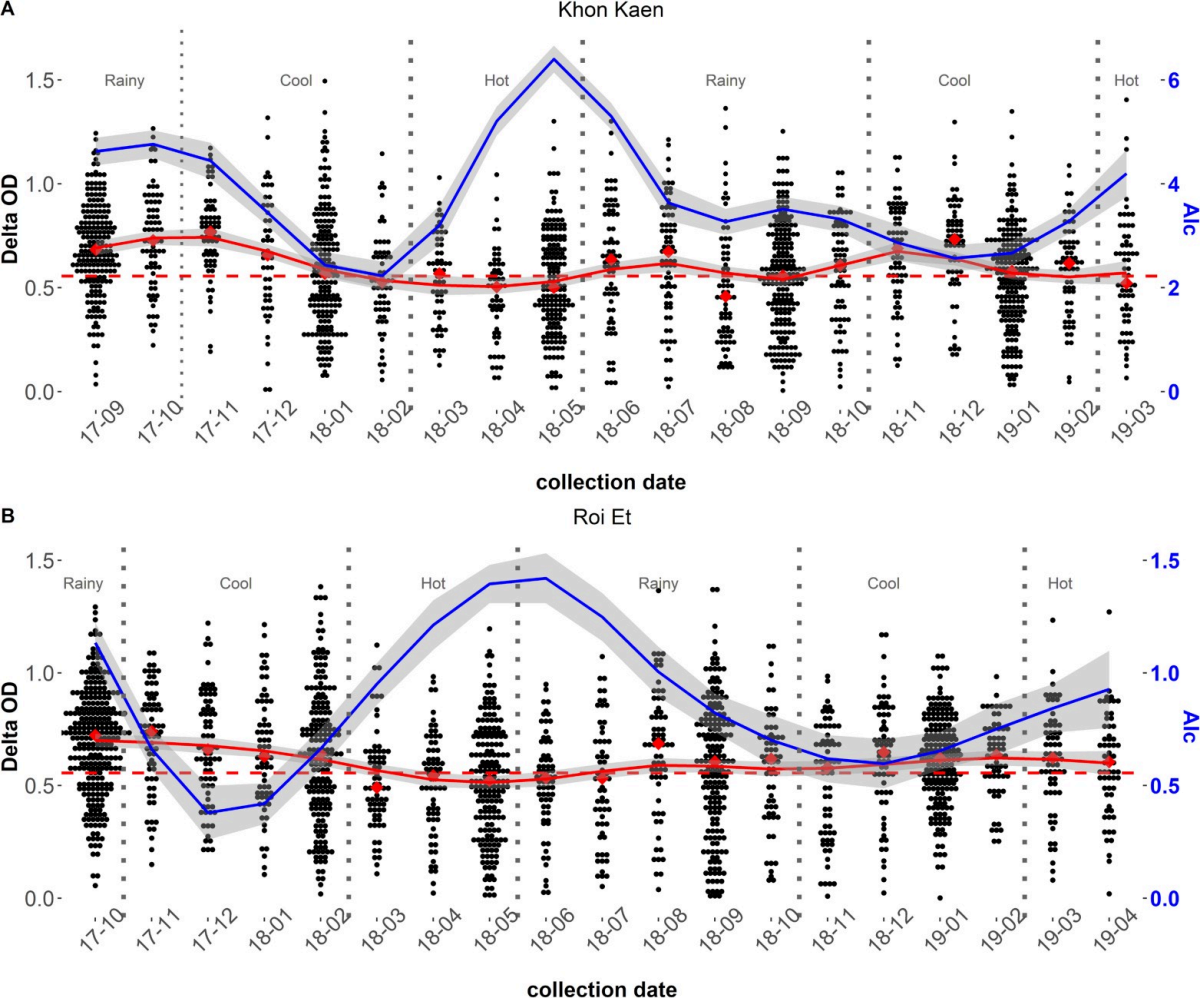
**Seasonal variations of the human IgG response to *Aedes* Nterm-34kDa salivary biomarker and the adult density *Aedes* Index (AI**_**c**_**), between September 2017 and April 2019 in Khon Kaen (A) and Roi Et (B) northeastern Thailand.** The dot plots represent the individual IgG immune response to the *Aedes* salivary peptide Nterm-34 kDa (ΔOD). The red diamonds represent the median response during each survey. The solid red lines represent the means and the grey shaded areas represent the confidence interval of the IgG response to the salivary biomarker. The red dashed horizontal lines represent the specific immune threshold TR. The solid blue lines represent the means and the grey shaded areas represent the 95% confidence interval respectively, for the AIc at the cluster level.

In both cities, *Aedes* density (AI_c_) strongly increased in May-June period corresponding to the end of the hot season and the beginning of the rainy season (Fig 3). Notably, the human IgG response (ΔOD) increased a few weeks after the measured peak of mosquito density. Additionally, the ΔOD decreased from the cool season until the hot season while the mosquito densities were reduced during the rainy season with numbers rebounding during the hot season. Collectively, the results indicated a lagged positive association between *Aedes* abundance and human exposure to *Aedes* bites. Indeed, previous studies on malaria vectors showed that the time-lag for human immune response was between three- to four- weeks after the vector bites [64]. Additionally, univariate analysis of the intensity of MEI indicated a positive association between the intensity of the human Ab response and the density of adult *Aedes* collected the month before the blood spot collection (S3 Table).

### Correlations between vector infestation, vector infectivity and human exposure risk to Aedes bites

**Fig 4:**
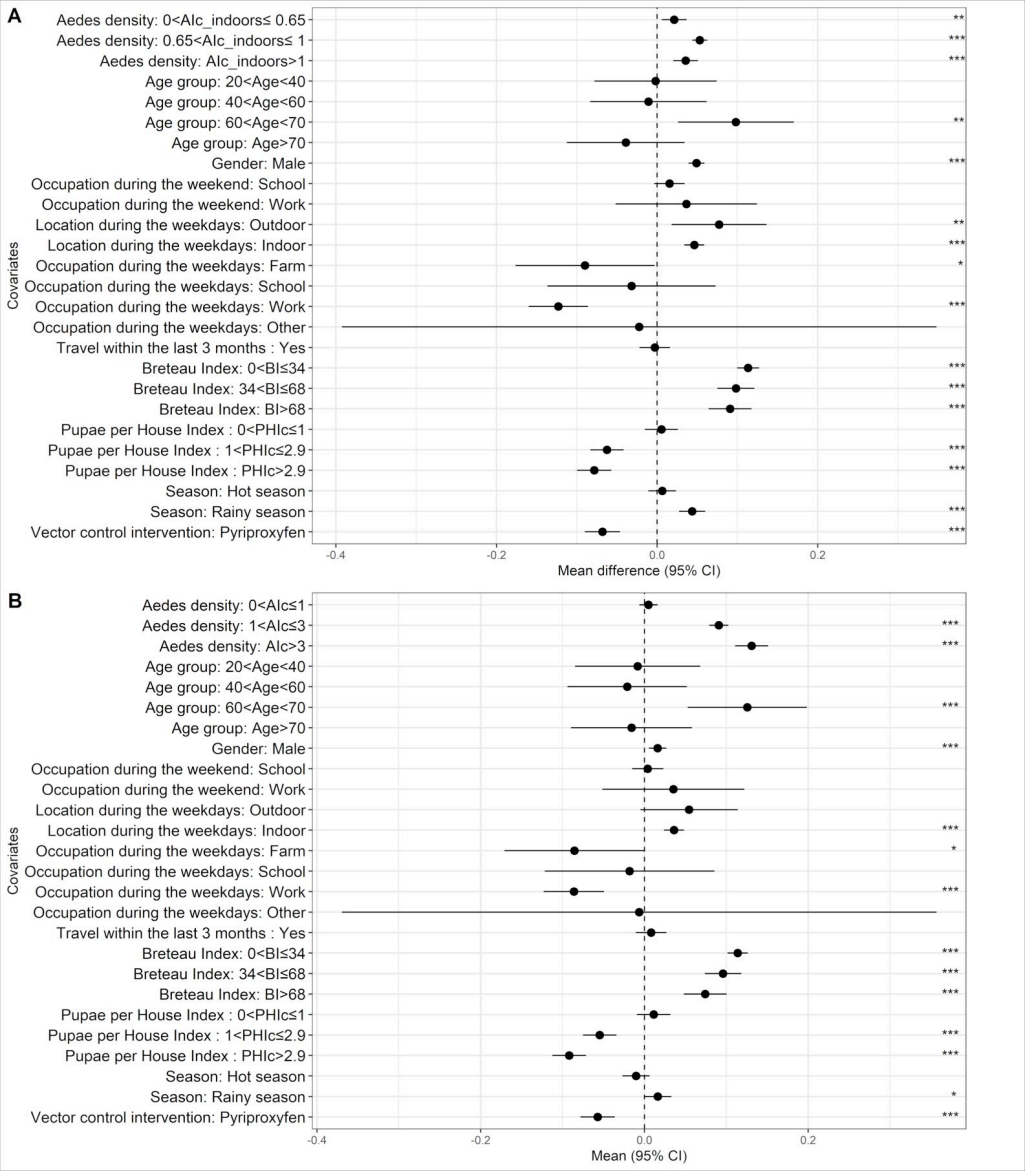
Multivariate analysis of MEI, human immune response to the Nterm-34 kDa salivary. (A) Adult *Aedes* indoors index only multivariate model. (B) Adult *Aedes* multivariate model.

Multivariate analysis was performed on a total of 539 individuals, with complete data, including 378 individuals followed-up every four months, with an average number of 2.63 visits per person. Additionally, a sub-sample of 161 individuals, followed-up every month with an average number of 12 visits per person were included in the analysis. The models showed a strong positive correlation between the MEI and the *Aedes* adult density at the cluster level when compared to the absence of *Aedes* for both the total adult AI_c_ (Fig 4B and Table 4, mean difference in MEI 0.091, p<0.0001, and 0.131, p<0.0001 for medium and high level of infestation, respectively) and the adult indoor density AI_in_c_ (Fig 4A and Table 4, difference in mean MEI of 0.021, p<0.007, 0.053, p<0.0001 and 0.037, p<0.0001 for low, medium and high levels of infestation, respectively). There was a significant positive association between the individual immune response and the three categories of *Aedes* intensity (low, medium and high), compared with the reference (no *Aedes)*, when considering adult mosquitoes collected indoors (p<0.05).

**Table 4:** Multivariate analysis of MEI, human immune response to Nterm-34 kDa salivary peptide.

		**AI**_**c**_ **(indoors and outdoors)**		**AI**_**c**_ **indoors only**	
		**Mean difference** ^a^	**P**	**Mean difference** ^a^	**P**
***Aedes* density**^**c**^			< .0001^b^		< .0001^b^
	No *Aedes*	**Reference**		**Reference**	
	Low	0.005	0.386	**0.021**	0.007
	Medium	**0.091**	< .0001	**0.053**	< .0001
	High	**0.131**	< .0001	**0.037**	< .0001
**Age,y**			< .0001^b^		< .0001^b^
	Age 5–19 y	**Reference**		**Reference**	
	Age 20–39 y	-0.008	0.833	0.003	0.941
	Age 40–59 y	-0.021	0.572	-0.009	0.812
	Age 60–69 y	**0.126**	0.001	**0.102**	0.007
	Age ≥70y	-0.016	0.676	-0.039	0.313
**Gender**			< .0001^b^		0.250^b^
	Female	**Reference**		Reference	
	Male	**0.016**	0.003	**0.048**	< .0001
**Occupation during the weekdays**			0.002^b^		0.028^b^
	Home	**Reference**		**Reference**	
	Work away from home	**-0.086**	< .0001	**-0.124**	< .0001
	School	-0.018	0.732	-0.031	0.568
	Farm	**-0.086**	0.050	**-0.088**	0.049
	Other	-0.006	0.972	-0.018	0.923
**Occupation during the weekends**			< .0001^b^		< .0001^b^
	Home	**Reference**		**Reference**	
	School	0.004	0.685	0.015	0.128
	Work	0.035	0.425	0.035	0.446
**Location during weekdays**			< .0001^b^		< .0001^b^
	Indoors and outdoors	**Reference**		**Reference**	
	Indoors	**0.036**	< .0001	**0.047**	**< .0001**
	Outdoors	0.055	0.0723	**0.077**	**0.012**
**Travel overall**			< .0001^b^		< .0001^b^
	No	**Reference**		**Reference**	
	Yes	0.008	0.393	-0.004	0.657
**Breteau Index**			< .0001^b^		< .0001^b^
	0	**Reference**		**Reference**	
	0–34	**0.114**	< .0001	**0.114**	< .0001
	34–68	**0.096**	< .0001	**0.098**	< .0001
	>68	**0.074**	< .0001	**0.091**	< .0001
**PHI_c_**			< .0001^b^		< .0001^b^
	0	**Reference**		**Reference**	
	0–1	0.011	0.281	0.006	0.689
	1–2.9	**-0.055**	< .0001	**-0.060**	< .0001
	>2.9	**-0.092**	< .0001	**-0.078**	< .0001
**Season**			0.003^b^		< .0001^b^
	Cool	**Reference**		**Reference**	
	Hot	-0.010	0.2258	0.006	0.474
	Rainy	0.016	0.054	**0.044**	< .0001
**Vector control intervention**			< .0001^b^		< .0001^b^
	Control	**Reference**		**Reference**	
	Pyriproxyfen	**-0.057**	< .0001	**-0.068**	< .0001

Analyses adjusted for rain, temperature maximum and cluster variables, in addition to the other specified variables. The difference in mean MEI in bold is significant at 0.05.

^a^ Defined as the difference between each class and the reference category

^b^ Likelihood ratio test to assess the global effect of the variable.

^c^ Adult density categories: 0–1, 1.1–3, and > 3 for AI_c_ class, low, medium and high, respectively. 0–0.65, 0.66–1 and >1 for AI_in_c_ *Aedes* female collected indoors categories low, medium and high, respectively.

In contrast, no clear relationships were noted between MEI and vector DENV infection at the cluster level (Table 5, p = 0.671) nor at the household level (Table 5, p = 0.764). Based on these study findings, the intensity of the immune response to *Aedes* bite exposure was not associated with a higher risk of being bitten by a DENV-infected vector (Table 5).

**Table 5:** Multivariate mixed linear model of human immune response to Nterm-34kDa *Aedes* salivary peptide or MEI and the presence of DENV infected *Aedes* in the cluster.

		**Cluster level**		**Household level**	
		**Mean difference** ^a^	**P**	**Mean difference** ^a^	**P**
**DENV infected *Aedes***			0.003^b^		0.050^b^
	0	**Reference**		**Reference**	
	> 0	0.012	0.671	-0.015	0.764

Analyses were adjusted for age, gender, travel history, BI_c,_ PHI_c_, season, and cluster variables, in addition to the other specified variables. The difference in mean MEI immune response in bold are significant at 0.05.

^a^ Defined as the difference between each class and the reference categories.

^b^ Likelihood ratio test to assess the global effect of the variable.

### Demographic, social, operational and climatic factors associated with human exposure risk to Aedes bites

For both models exploring AI_c_ and AI_in_c_, using univariate analysis (S3 Table), all covariates (except “remain at home during the last 7 days”) were retained in the analysis. MEI differed according to age (p<0.0001), sex (p<0.0001), season (p = 0.003), vector control intervention (p<0.0001) and human occupation (p<0.0001) (Fig 4 and Table 4). The 60–69 years old age group had higher levels of antibody response to *Aedes* bites compared to other classes (Table 4 and Fig 4, p<0.001). Additionally, being male was associated with a higher risk of having had *Aedes* bites (p = 0.003 and p<0.0001) in both models. Interestingly, people spending greater time preferentially indoors during weekdays had higher levels of IgG response to salivary peptide than people spending time both indoors and outdoors (Table 4 and Fig 4, difference in MEI mean 0.036, p<0.0001 and 0.047, p<0.0001 for total *Aedes* density and indoor *Aedes* density, respectively).

Several entomological indices of immature stages were significantly correlated to the MEI. The Breteau Index was positively associated with IgG seroprevalence to the Nterm-34 kDa, although the strength of the association seemed to saturate at higher levels. Interestingly, the Pupae per House Index (PHI_c_) at the cluster level was negatively correlated with the MEI (Table 4 and Fig 4, p<0.0001). In both models, the presence of the trial vector control intervention was associated with a decreased level of antibody response against *Aedes* bites (Table 4 and Fig 4, difference in MEI mean -0.057 at p<0.0001 and -0.068 at p<0.0001 for the AI_c_ and the AI_in_c_ models respectively). Regarding climatic factors, the rainy season was positively associated with MEI in both models.

## Discussion

This study highlights a strong positive relationship between the intensity of human IgG response against the *Aedes* salivary peptide Nterm-34kDa and adult *Aedes* population densities in association with humans in northeastern Thailand. A clear gradient response between the MEI and adult vector density indicated that individuals exhibiting higher antibody response to the *Aedes* salivary peptide were located in areas with higher risk of potential dengue vector bites. This study corroborates previous work [35–41] showing that the serological biomarker represents a promising surveillance tool to assess small-scale variations in human exposure risk to *Aedes* bites in dengue endemic settings. Although studied for malaria vectors [34], this is the first longitudinal study combining both entomological and immunological endpoints investigating *Aedes* vectors and virus transmission. Further investigations are needed to address the kinetics of human immune response to *Aedes* salivary proteins, in particular the delay between bite exposure, and the production and waning of IgG titers.

This study showed that the human-mosquito contact is influenced by human behavioral characteristics, socio-demographic conditions, climatic factors, and trial vector control interventions associated with dengue transmission risk as previously demonstrated [8,19,56]. The relationship between human dengue infections and the intensity of the human-antibody response to *Aedes* bites could not be ascertained because incident dengue cases were not detected in the study participants during the time of longitudinal follow-up. Further analysis is on-going to confirm the observation of the apparent lack (or very low) transmission during the study period (to be reported elsewhere). In a recent case-control study conducted in northeastern Thailand (conducted by this study team), neither the adult mosquito abundance at the household level nor the degree of human exposure to *Aedes* bites was correlated with a higher odds of acquiring dengue infection [56]. Although consistent with some previous results in Southeast Asia [43,56], the small sample size of DENV-positive *Aedes* might explain the lack of significance between the human-*Aedes* exposure and the risk of DENV infected bites seen in this study. This highlights dengue virus transmission is both a multi-factorial and a complex affair that varies over time and space, and the relationship between vector density and virus transmission is dynamic and thus might not be adequately or accurately characterized through standard methods of entomological monitoring.

These findings show that the MEI was significantly associated with the season and prevailing climatic factors. The proportion of immune responders to *Aedes* bites was higher during the rainy season than the drier months of the year, corresponding to the period of greater adult vector densities. This is probably explained by the dramatic increase in most entomological indices during this period of the year where the number of suitable larval habitats increases and adult survival (longevity) is presumably enhanced [15,65]. Similar results were reported in Benin, where the overall anti-saliva antibody response in children increased during the rainy season [42]. A recent study in Cote d’Ivoire highlighted a strong relationship between human mosquito exposure, season and agricultural practices [66]. Specific IgG responses remained high during both seasons in villages associated with intensive agricultural compared to villages lacking agricultural practices. The authors suggest that the presence of rubber and oil palm plantations, by providing a suitable environment for the presence of *Aedes* vector species maintained a high level of human exposure to *Aedes* mosquito bites regardless of annual seasonal changes.

Interestingly, the present study also suggests correlations between the MEI and *Aedes* immature-based indices, although the association appeared weaker compared to adult measures. The Breteau Index was associated with higher levels of antibody response against *Aedes* bites but was not gradient-dependent. In contrast, the pupae per house index was negatively associated with the MEI. This result might seem contradictory; however, that under natural field conditions, larvae and pupae development rates are strongly influenced by climatic factors, particularly ambient temperature and rainfall patterns, as well as density-dependent factors of immature stages affecting resource competition [67–69]. Additionally, the presence of larval stages in an aquatic habitat can inhibit further egg hatching [70]. Therefore, a decrease in human immune response to *Aedes* bites could be the reflection of the cyclic fluctuations between successive adult population densities influenced by site-specific immature mosquito densities.

The MEI varied according to individual characteristics, such as gender, age, and occupation. Interestingly, older people presented higher risk for mosquito bites than the younger population. Similarly, being a male was associated with a higher exposure level to *Aedes* bites. Similar results were found with *Anopheles* exposure and malaria transmission in Thailand, where males were at higher risk than females, mainly due to differences in behavior and occupational exposure [37]. Nevertheless, these results have to be viewed with caution as the majority of the participants in the present study were female and the median age of the cohort was 64 in KK and 61 in RE, which may have biased the outcomes. Indeed, the median age of the cohort reflects the lack of representation of the younger population, which are presumed more active (mobile) than older individuals. Our findings also showed that individuals spending the majority of time indoors were associated with a higher exposure to *Aedes* bites than those spending time more equally either indoors and out. An explanation is that *Ae*. *aegypti* is a well-adapted species for resting and breeding inside dwellings, and is more typically found indoors [22,23]. This is also supported by the level of significance of human-exposure risk using the *Aedes* indoor index. The risk of biting (i.e., transmission) inside a dwelling appears particularly important and helps explain why insecticide-treated curtains and targeted indoor residual spraying were highly effective against *Ae*. *aegypti* for the control and prevention of dengue outbreaks in Mexico and Australia [46,71].

This study suggests that the salivary biomarker is sensitive enough to detect small scale variations in human exposure to *Aedes* bites over time, in particular during a vector control intervention. The human IgG levels were significantly lower in treated clusters compared to the control clusters. These findings would suggest an appreciable impact of pyriproxyfen treatment on the density of *Aedes* adult populations. Similar results were observed in La Réunion, where vector control intervention combining *Aedes* larval habitat source reduction and insecticide space spray against adult mosquitoes was associated with a significant decrease in human antibody response against *Ae*. *albopictu*s bites [41,63]. Investigations are on-going in Thailand to assess the entomological and epidemiological impact of pyriproxyfen intervention in the study area [48,72].

This study represents an important step toward the validation of using the *Aedes* salivary peptide Nterm-34kDa as a proxy measure to assess *Aedes* infestation levels and human-mosquito exposure risk in a dengue endemic area. Although promising results are described, the use of the Nterm-34 kDa as a surveillance indicator for estimating dengue transmission risk requires further investigations including other geographical and transmission settings.

## Supporting information

S1 Table Defined variables. (DOCX)

S2 Table Comparison of proportion of immune responders and entomological indices between Khon Kaen and Roi Et provinces using Chi square test and ANOVA. (DOCX)

S3 Table Univariate analysis of the human antibody response to the Aedes salivary biomarker Nterm-34 kDa. (DOCX)

## References

[1] Bhatt S, Gething P, Brady O, Messina J, Farlow A, Moyes C, et al. The global distribution and burden of dengue. Nature. 2013. doi: 10.1038/nature12060 23563266 PMC3651993

[2] World Health Organization. Dengue guidelines for diagnosis, treatment, prevention and control Geneva: WHO; 2009. 23762963

[3] Hammon WM. Dengue hemorrhagic fever—do we know its cause? Am J Trop Med Hyg. 1973;22(1):82–91. doi: 10.4269/ajtmh.1973.22.82 4567852

[4] Bureau of Epidemiology, Thailand MoPH. Annual epidemiological surveillance report 2018 2019. Available from: https://apps.doe.moph.go.th/boeeng/download/AW_Annual_Mix%206212_14_r1.pdf.

[5] Shepard DS, Undurraga EA, Halasa YA. Economic and disease burden of dengue in Southeast Asia. PLoS Negl Trop Dis. 2013;7(2):e2055. doi: 10.1371/journal.pntd.0002055 23437406 PMC3578748

[6] Limkittikul K, Brett J, L’Azou M. Epidemiological trends of dengue disease in Thailand (2000–2011): a systematic literature review. PLoS Negl Trop Dis. 2014;8(11):e3241. doi: 10.1371/journal.pntd.0003241 25375766 PMC4222696

[7] Bureau of Emerging Infectious Disease. Thailand’s National Strategic Plan For Emerging Infectious Disease Preparedness, Prevention and Response 2013–2016. In: Department of Disease Control MoPH, editor. Nonthaburi, Thailand: the War Veterans Organization of Thailand Under Royal Patronage of His Majesty the King; 2013. p. 100.

[8] Bowman LR, Runge-Ranzinger S, McCall PJ. Assessing the relationship between vector indices and dengue transmission: a systematic review of the evidence. PLoS Negl Trop Dis. 2014;8(5):e2848. doi: 10.1371/journal.pntd.0002848 24810901 PMC4014441

[9] Chadee DD. Dengue cases and Aedes aegypti indices in Trinidad, West Indies. Acta Trop. 2009;112(2):174–80. doi: 10.1016/j.actatropica.2009.07.017 19632189

[10] Sanchez L, Vanlerberghe V, Alfonso L, Marquetti Mdel C, Guzman MG, Bisset J, et al. Aedes aegypti larval indices and risk for dengue epidemics. Emerg Infect Dis. 2006;12(5):800–6. doi: 10.3201/eid1205.050866 16704841 PMC3374431

[11] Chadee DD, Williams FL, Kitron UD. Impact of vector control on a dengue fever outbreak in Trinidad, West Indies, in 1998. Trop Med Int Health. 2005;10(8):748–54. doi: 10.1111/j.1365-3156.2005.01449.x 16045461

[12] Ooi EE, Goh KT, Gubler DJ. Dengue prevention and 35 years of vector control in Singapore. Emerg Infect Dis. 2006;12(6):887–93. doi: 10.3201/10.3201/eid1206.051210 16707042 PMC3373041

[13] Romero-Vivas CM, Falconar AK. Investigation of relationships between Aedes aegypti egg, larvae, pupae, and adult density indices where their main breeding sites were located indoors. J Am Mosq Control Assoc. 2005;21(1):15–21. doi: 10.2987/8756-971X(2005)21[15:IORBAA]2.0.CO;2 15825756

[14] Focks D. A review of entomological sampling methods and indicators for dengue vectors. WHO-TDR. 2004. doi: 10.1603/0022-2585-41.6.1123

[15] Focks DA, Haile DG, Daniels E, Mount GA. Dynamic life table model for Aedes aegypti (Diptera: Culicidae): analysis of the literature and model development. J Med Entomol. 1993;30(6):1003–17. doi: 10.1093/jmedent/30.6.1003 8271242

[16] Roiz D, Wilson AL, Scott TW, Fonseca DM, Jourdain F, Muller P, et al. Integrated Aedes management for the control of Aedes-borne diseases. PLoS Negl Trop Dis. 2018;12(12):e0006845. doi: 10.1371/journal.pntd.0006845 30521524 PMC6283470

[17] Focks DA, Brenner RJ, Hayes J, Daniels E. Transmission thresholds for dengue in terms of Aedes aegypti pupae per person with discussion of their utility in source reduction efforts. Am J Trop Med Hyg. 2000;62(1):11–8. 10761719

[18] Focks DA, Chadee DD. Pupal survey: an epidemiologically significant surveillance method for Aedes aegypti: an example using data from Trinidad. Am J Trop Med Hyg. 1997;56(2):159–67. doi: 10.4269/ajtmh.1997.56.159 9080874

[19] Parra MCP, Favaro EA, Dibo MR, Mondini A, Eiras AE, Kroon EG, et al. Using adult Aedes aegypti females to predict areas at risk for dengue transmission: A spatial case-control study. Acta Trop. 2018;182:43–53. doi: 10.1016/j.actatropica.2018.02.018 29462598

[20] Lau SM, Chua TH, Sulaiman WY, Joanne S, Lim YA, Sekaran SD, et al. A new paradigm for Aedes spp. surveillance using gravid ovipositing sticky trap and NS1 antigen test kit. Parasit Vectors. 2017;10(1):151. doi: 10.1186/s13071-017-2091-y 28327173 PMC5361725

[21] Harrington LC, Fleisher A, Ruiz-Moreno D, Vermeylen F, Wa CV, Poulson RL, et al. Heterogeneous feeding patterns of the dengue vector, Aedes aegypti, on individual human hosts in rural Thailand. PLoS Negl Trop Dis. 2014;8(8):e3048. doi: 10.1371/journal.pntd.0003048 25102306 PMC4125296

[22] Scott TW, Chow E, Strickman D, Kittayapong P, Wirtz RA, Lorenz LH, et al. Blood-feeding patterns of Aedes aegypti (Diptera: Culicidae) collected in a rural Thai village. J Med Entomol. 1993;30(5):922–7. doi: 10.1093/jmedent/30.5.922 8254642

[23] Scott TW, Clark GG, Lorenz LH, Amerasinghe PH, Reiter P, Edman JD. Detection of multiple blood feeding in Aedes aegypti (Diptera: Culicidae) during a single gonotrophic cycle using a histologic technique. J Med Entomol. 1993;30(1):94–9. doi: 10.1093/jmedent/30.1.94 8433350

[24] Ortega-López LD, Pondeville E, Kohl A, León R, Betancourth MP, Almire F, et al. The mosquito electrocuting trap as an exposure-free method for measuring human-biting rates by Aedes mosquito vectors. Parasites & vectors. 2020;13(1):31–. doi: 10.1186/s13071-020-3887-8 31941536 PMC6961254

[25] Bazin M, Williams CR. Mosquito traps for urban surveillance: collection efficacy and potential for use by citizen scientists. J Vector Ecol. 2018;43(1):98–103. doi: 10.1111/jvec.12288 29757507

[26] Barnard DR, Dickerson CZ, Murugan K, Xue RD, Kline DL, Bernier UR. Measurement of landing mosquito density on humans. Acta Trop. 2014;136:58–67. doi: 10.1016/j.actatropica.2014.04.019 24769003

[27] Doucoure S, Drame PM. Salivary biomarkers in the control of mosquito-borne diseases. Insects. 2015;6(4):961–76. doi: 10.3390/insects6040961 26593952 PMC4693181

[28] Poinsignon A, Cornelie S, Mestres-Simon M, Lanfrancotti A, Rossignol M, Boulanger D, et al. Novel peptide marker corresponding to salivary protein gSG6 potentially identifies exposure to Anopheles bites. PLoS One. 2008;3(6):e2472. doi: 10.1371/journal.pone.0002472 18575604 PMC2427200

[29] Lombardo F, Ronca R, Rizzo C, Mestres-Simon M, Lanfrancotti A, Curra C, et al. The Anopheles gambiae salivary protein gSG6: an anopheline-specific protein with a blood-feeding role. Insect Biochem Mol Biol. 2009;39(7):457–66. doi: 10.1016/j.ibmb.2009.04.006 19442731 PMC3740408

[30] Wasinpiyamongkol L, Patramool S, Luplertlop N, Surasombatpattana P, Doucoure S, Mouchet F, et al. Blood-feeding and immunogenic Aedes aegypti saliva proteins. Proteomics. 2010;10(10):1906–16. doi: 10.1002/pmic.200900626 19882664

[31] Fontaine A, Pascual A, Orlandi-Pradines E, Diouf I, Remoué F, Pagès F, et al. Relationship between exposure to vectors bites and antibody response to mosquito salivary gland extracts. PLoS ONE. 2011;6(12). doi: 10.1371/journal.pone.0029107 22195000 PMC3237593

[32] Remoue F, Cisse B, Ba F, Sokhna C, Herve JP, Boulanger D, et al. Evaluation of the antibody response to Anopheles salivary antigens as a potential marker of risk of malaria. Trans R Soc Trop Med Hyg. 2006;100(4):363–70. doi: 10.1016/j.trstmh.2005.06.032 16310235

[33] Orlandi-Pradines E, Almeras L, Denis de Senneville L, Barbe S, Remoue F, Villard C, et al. Antibody response against saliva antigens of Anopheles gambiae and Aedes aegypti in travellers in tropical Africa. Microbes and infection. 2007;9(12–13):1454–62. doi: 10.1016/j.micinf.2007.07.012 17913537

[34] Ya-Umphan P, Cerqueira D, Parker DM, Cottrell G, Poinsignon A, Remoue F, et al. Use of an Anopheles salivary biomarker to assess malaria transmission risk along the Thailand-Myanmar border. J Infect Dis. 2017;215(3):396–404. doi: 10.1093/infdis/jiw543 27932615 PMC5853934

[35] Poinsignon A, Cornelie S, Ba F, Boulanger D, Sow C, Rossignol M, et al. Human IgG response to a salivary peptide, gSG6-P1, as a new immuno-epidemiological tool for evaluating low-level exposure to Anopheles bites. Malar J. 2009;8:198. doi: 10.1186/1475-2875-8-198 19674487 PMC2733152

[36] Marie A, Ronca R, Poinsignon A, Lombardo F, Drame PM, Cornelie S, et al. The Anopheles gambiae cE5 salivary protein: a sensitive biomarker to evaluate the efficacy of insecticide-treated nets in malaria vector control. Microbes and infection. 2015;17(6):409–16. doi: 10.1016/j.micinf.2015.01.002 25637950

[37] Ya-Umphan P, Cerqueira D, Cottrell G, Parker DM, Fowkes FJI, Nosten F, et al. Anopheles salivary biomarker as a proxy for estimating Plasmodium falciparum malaria exposure on the Thailand-Myanmar border. Am J Trop Med Hyg. 2018;99(2):350–6. doi: 10.4269/ajtmh.18-0081 29869601 PMC6090370

[38] Londono-Renteria B, Cardenas JC, Cardenas LD, Christofferson RC, Chisenhall DM, Wesson DM, et al. Use of anti-Aedes aegypti salivary extract antibody concentration to correlate risk of vector exposure and dengue transmission risk in Colombia. PLoS One. 2013;8(12):e81211. doi: 10.1371/journal.pone.0081211 24312537 PMC3846924

[39] Doucoure S, Mouchet F, Cournil A, Le Goff G, Cornelie S, Roca Y, et al. Human antibody response to Aedes aegypti saliva in an urban population in Bolivia: a new biomarker of exposure to dengue vector bites. Am J Trop Med Hyg. 2012;87(3):504–10. doi: 10.4269/ajtmh.2012.11-0477 22848099 PMC3435356

[40] Mathieu-Daude F, Claverie A, Plichart C, Boulanger D, Mphande FA, Bossin HC. Specific human antibody responses to Aedes aegypti and Aedes polynesiensis saliva: A new epidemiological tool to assess human exposure to disease vectors in the Pacific. PLoS Negl Trop Dis. 2018;12(7):e0006660. doi: 10.1371/journal.pntd.0006660 30040826 PMC6075770

[41] Doucoure S, Mouchet F, Cornelie S, Drame PM, D’Ortenzio E, DeHecq JS, et al. Human antibody response to Aedes albopictus salivary proteins: a potential biomarker to evaluate the efficacy of vector control in an area of chikungunya and dengue virus transmission. Biomed Res Int. 2014;2014:746509. doi: 10.1155/2014/746509 24822216 PMC4005104

[42] Elanga Ndille E, Doucoure S, Damien G, Mouchet F, Drame PM, Cornelie S, et al. First attempt to validate human IgG antibody response to Nterm-34kDa salivary peptide as biomarker for evaluating exposure to Aedes aegypti bites. PLoS Negl Trop Dis. 2012;6(11):e1905. doi: 10.1371/journal.pntd.0001905 23166852 PMC3499371

[43] Elanga Ndille E, Dubot-Peres A, Doucoure S, Mouchet F, Cornelie S, Sidavong B, et al. Human IgG antibody response to Aedes aegypti Nterm-34 kDa salivary peptide as an indicator to identify areas at high risk for dengue transmission: a retrospective study in urban settings of Vientiane city, Lao PDR. Trop Med Int Health. 2014;19(5):576–80. doi: 10.1111/tmi.12280 24641205

[44] Sagna AB, Kassie D, Couvray A, Adja AM, Hermann E, Riveau G, et al. Spatial assessment of contact between humans and Anopheles and Aedes mosquitoes in a medium-sized African urban setting, using salivary antibody-based biomarkers. J Infect Dis. 2019;220(7):1199–208. doi: 10.1093/infdis/jiz289 31152664

[45] Bowman L, Donegan S, McCall P. Is dengue vector control deficient in effectiveness or evidence?: Systematic review and meta-analysis. PLoS Negl Trop Dis. 2016;10(3). doi: 10.1371/journal.pntd.0004551 26986468 PMC4795802

[46] Erlanger TE, Keiser J, Utzinger J. Effect of dengue vector control interventions on entomological parameters in developing countries: a systematic review and meta-analysis. Med Vet Entomol. 2008;22(3):203–21. doi: 10.1111/j.1365-2915.2008.00740.x 18816269

[47] Overgaard HJ, Pientong C, Thaewnongiew K, Bangs MJ, Ekalaksananan T, Aromseree S, et al. Correction to: Assessing dengue transmission risk and a vector control intervention using entomological and immunological indices in Thailand: study protocol for a cluster-randomized controlled trial. Trials. 2018;19(1):703. doi: 10.1186/s13063-018-3110-9 30583732 PMC6304772

[48] Overgaard HJ, Pientong C, Thaewnongiew K, Bangs MJ, Ekalaksananan T, Aromseree S, et al. Assessing dengue transmission risk and a vector control intervention using entomological and immunological indices in Thailand: study protocol for a cluster-randomized controlled trial. Trials. 2018;19(1):122. doi: 10.1186/s13063-018-2490-1 29458406 PMC5819278

[49] Thailand administrative boundaries common operational database [Internet]. United Nation Office for the Coordination of Humanitarian Affairs. 2019 [cited May 5, 2020]. Available from: https://data.humdata.org/dataset/thailand-administrative-boundaries.

[50] Roland JW, Hooff GP. State-of-the-art dried blood spot analysis: an overview of recent adavnces and future trends. Bioanalysis. 2013;5(17). 10.4155/bio.13.175 23962253

[51] Rueda LM. Pictorial keys for the identification of mosquitoes (Diptera: Culicidae) associated with dengue virus transmission. Auckland: Magnolia Press; 2004.

[52] Bangs MJ, Focks DA. Abridged pupa identification key to the common container-breeding mosquitoes in urban Southeast Asia. J Am Mosq Control Assoc. 2006;22(3):565–72. doi: 10.2987/8756-971X(2006)22[565:APIKTT]2.0.CO;2 17067066

[53] Vazquez-Prokopec GM, Galvin WA, Kelly R, Kitron U. A new, cost-effective, battery-powered aspirator for adult mosquito collections. J Med Entomol. 2009;46(6):1256–9. doi: 10.1603/033.046.0602 19960668 PMC2800949

[54] Lanciotti RS, Calisher CH, Gubler DJ, Chang GJ, Vorndam AV. Rapid detection and typing of dengue viruses from clinical samples by using reverse transcriptase-polymerase chain reaction. Journal of clinical microbiology. 1992;30(3):545–51. doi: 10.1128/JCM.30.3.545-551.1992 1372617 PMC265106

[55] Thai Meteorological Department. [Available from: https://www.tmd.go.th/en/.

[56] Fustec B, Phanitchat T, Hoq MI, Aromseree S, Pientong C, Thaewnongiew K, et al. Complex relationships between Aedes vectors, socio-economics and dengue transmission—lessons learned from a case-control study in northeastern Thailand. PLoS Negl Trop Dis. 2020. doi: 10.1371/journal.pntd.0008703 33001972 PMC7553337

[57] Kraemer MUG, Sinka ME, Duda KA, Mylne AQN, Shearer FM, Barker CM, et al. The global distribution of the arbovirus vectors Aedes aegypti and Ae. albopictus. eLife. 2015;4:e08347–e. doi: 10.7554/eLife.08347 26126267 PMC4493616

[58] Ripley BD, Venables WN. Modern applied statistics with S. New York: Springer; 2002. Available from: http://www.stats.ox.ac.uk/pub/MASS4.

[59] Fox J. The R Commander: a basic statistics graphical user interface to R. Journal of Statistical Software. 2005;14(9):42.

[60] Pinheiro J, Bates D, DebRoy S, Sarkar D, R Core Team. nlme: Linear and nonlinear mixed effect models. 2020.

[61] Wickham H. ggplot2: elegant graphics for data analysis: Springer-Verlag New York; 2016.

[62] Kassambara A. ggpubr: ’ggplot2’ Based publication ready plots. R package version 0.2. 2018.

[63] Elanga Ndille E, Doucoure S, Poinsignon A, Mouchet F, Cornelie S, D’Ortenzio E, et al. Human IgG antibody response to Aedes Nterm-34kDa salivary peptide, an epidemiological tool to assess vector control in chikungunya and dengue transmission area. PLoS Negl Trop Dis. 2016;10(12):e0005109. doi: 10.1371/journal.pntd.0005109 27906987 PMC5131890

[64] Drame PM, Poinsignon A, Besnard P, Cornelie S, Le Mire J, Toto JC, et al. Human antibody responses to the Anopheles salivary gSG6-P1 peptide: a novel tool for evaluating the efficacy of ITNs in malaria vector control. PLoS One. 2010;5(12):e15596. doi: 10.1371/journal.pone.0015596 21179476 PMC3001874

[65] Brady OJ, Johansson MA, Guerra CA, Bhatt S, Golding N, Pigott DM, et al. Modelling adult Aedes aegypti and Aedes albopictus survival at different temperatures in laboratory and field settings. Parasit Vectors. 2013;6:351. doi: 10.1186/1756-3305-6-351 24330720 PMC3867219

[66] Yobo CM, Sadia-Kacou CAM, Adja MA, Elanga-Ndille E, Sagna AB, Guindo-Coulibaly N, et al. Evaluation of human exposure to Aedes bites in rubber and palm cultivations using an immunoepidemiological biomarker. Biomed Res Int. 2018;2018:3572696. doi: 10.1155/2018/3572696 30175128 PMC6106716

[67] Focks DA, Haile DG, Daniels E, Mount GA. Dynamic life table model for Aedes aegypti (Diptera: Culicidae): Simulation results and validation. Journal of Medical Entomology. 1993;30(6):1018–28. doi: 10.1093/jmedent/30.6.1018 8271243

[68] Legros M, Magori K, Morrison AC, Xu C, Scott TW, Lloyd AL, et al. Evaluation of location-specific predictions by a detailed simulation model of Aedes aegypti populations. PloS one. 2011;6(7):e22701–e. doi: 10.1371/journal.pone.0022701 21799936 PMC3143176

[69] Magori K, Legros M, Puente ME, Focks DA, Scott TW, Lloyd AL, et al. Skeeter Buster: a stochastic, spatially explicit modeling tool for studying Aedes aegypti population replacement and population suppression strategies. PLoS neglected tropical diseases. 2009;3(9):e508–e. doi: 10.1371/journal.pntd.0000508 19721700 PMC2728493

[70] Aznar V, Otero M, De Majo M, Fischer S, Solari H. Modeling the complex hatching and development of Aedes aegypti in temperate climates. Ecological Modelling. 2013;253:44–55.

[71] Vazquez-Prokopec GM, Montgomery BL, Horne P, Clennon JA, Ritchie SA. Combining contact tracing with targeted indoor residual spraying significantly reduces dengue transmission. Sci Adv. 2017;3(2):e1602024. doi: 10.1126/sciadv.1602024 28232955 PMC5315446

[72] Rochon J. Issues in adjusting for covariates arising post randomization in clinical trials. Drug Information Journal. 1999;33(4):1219–28.

