# Supplementary material for: Serological biomarker for assessing human exposure to Aedes mosquito bites during a randomized vector control intervention trial in northeastern Thailand
Source: PLoS Negl Trop Dis. 2021 May 27;15(5):e0009440. doi: 10.1371/journal.pntd.0009440 (PMC8189451; doi:10.1371/journal.pntd.0009440)
Supplement: S1 Table — (DOCX) [file pntd.0009440.s001.docx]

**Supplementary Table S1:** Defined variables

| **Variable** | **Definition / Formula** | ***Aedes* life stage** |
| --- | --- | --- |
| *Individual level* |  |  |
| MEI | MEI= ∆OD-TR  IgG response to Nterm 34 kDa salivary peptide | Adult |
| *Household level* |  |  |
| AI DENV+ | No female *Aedes* DENV infected/ No female *Aedes* tested per house | Adult |
| *Cluster level* |  |  |
| CI_c_ (%) | (No positive container/ total no of wet container) x 100 | Larvae & Pupae |
| HI_c_ (%) | (No positive house/ No households visited) x 100 | Larvae & Pupae |
| BI_c_ | No positive container x100/ No containers inspected / no house visited | Larvae & Pupae |
| AI_c_ | No of female adult *Aedes* collected/ No households visited | Adult |
| AI_in_c_ | No of adult female *Aedes* collected indoor/ No households visited | Adult |
| PHI_c_ | No of pupae collected/ No houses visited | Pupae |
| PPI_c_ | No of pupae collected/ per person/No households visited. | Pupae |
| AI_c_ DENV+ | No female *Aedes* DENV infected/ No female Aedes tested at the cluster level | Adult |
