# Supplementary material for: Serological biomarker for assessing human exposure to Aedes mosquito bites during a randomized vector control intervention trial in northeastern Thailand
Source: PLoS Negl Trop Dis. 2021 May 27;15(5):e0009440. doi: 10.1371/journal.pntd.0009440 (PMC8189451; doi:10.1371/journal.pntd.0009440)
Supplement: S2 Table — (DOCX) [file pntd.0009440.s002.docx]

**Supplementary Table S2.** Comparison of proportion of immune responders and entomological indices between Khon Kaen and Roi Et provinces using Chi square test and ANOVA.

|  | Khon Kaen | Roi Et | p-value |
| --- | --- | --- | --- |
| Proportion of immune responders | 57.8% | 60.5% | 0.08^a^ |
| CI_c_ (mean) | 16.4% | 4.1% | <0.0001^b^ |
| HI_c_ (mean) | 45.5% | 12.9% | <0.0001^b^ |
| BI_c_ (mean) | 61.2 | 13.3 | <0.0001^b^ |
| PHI_c_ (mean) | 0.63 | 0.84 | <0.0001^b^ |
| PPI_c_ (mean) | 0.19 | 0.26 | <0.0001^b^ |
| AI_c_ (mean) | 3.71 | 0.79 | <0.0001^b^ |
| AI_in_c_ (mean) | 1.00 | 0.68 | <0.0001^b^ |
| ^a^ tested by Chi square test  ^b^ tested by ANOVA | | | |
