# Supplementary material for: Serological biomarker for assessing human exposure to Aedes mosquito bites during a randomized vector control intervention trial in northeastern Thailand
Source: PLoS Negl Trop Dis. 2021 May 27;15(5):e0009440. doi: 10.1371/journal.pntd.0009440 (PMC8189451; doi:10.1371/journal.pntd.0009440)
Supplement: S3 Table — (DOCX) [file pntd.0009440.s003.docx]

**Supplementary Table S3:** Univariate analysis of human immune response to salivary peptide Nterm-34kDa.

|  |  | **Mean difference ^a^** | **p-value** |
| --- | --- | --- | --- |
| **Individual covariates** | | | |
| **Age, years** |  |  | <0.0001^b^ |
|  | Age 5-19 y | Reference |  |
|  | Age 20-39 y | 0.222 | 0.082 |
|  | Age 40-59 y | 0.171 | 0.159 |
|  | Age 60-69 y | 0.100 | 0.396 |
|  | Age ≥70y | **0.298** | 0.017 |
| **Gender** |  |  | <0.0001^b^ |
|  | Female | Reference |  |
|  | Male | **-0.022** | <0.0001 |
| **Occupation during the weekdays** |  |  | <0.0001^b^ |
|  | Home | Reference |  |
|  | Work away from home | **-0.782** | <0.0001 |
|  | School | -0.168 | 0.060 |
|  | Farming | -0.157 | 0.444 |
|  | Other | -0.150 | 0.853 |
| **Occupation during the weekends** |  |  | <0.0001^b^ |
|  | Home | Reference |  |
|  | School | **0.157** | <0.0001 |
|  | Work away from home | **-0.599** | <0.0001 |
|  | Other | -0.100 | 0.869 |
| **Location during weekdays** |  |  | <0.0001^b^ |
|  | Both indoors and outdoors | Reference |  |
|  | Indoors | **<.0001** | <0.0001 |
|  | Outdoors | **0.001** | 0.0014 |
| **Location during weekends** |  |  | <0.0001^b^ |
|  | Both indoors and outdoors | Reference |  |
|  | Indoors | **0.132** | <0.0001 |
|  | Outdoors | 0.090 | 0.7337 |
| **Travel within the last 14 days** |  |  | <0.0001^b^ |
|  | No | Reference |  |
|  | Yes | **0.082** | <0.0001 |
| **Travel within the last 3 months** |  |  | <0.0001^b^ |
|  | No | Reference |  |
|  | Yes | **-0.105** | <0.0001 |
| **Travel overall (recent)** |  |  | <0.0001^b^ |
|  | No | Reference |  |
|  | Yes | **0.155** | <0.0001 |
| **Household covariate** | | | |
| **DENV infected Aedes**  **(AI DENV+)** |  |  | 0.0002^b^ |
|  | 0 | Reference |  |
|  | >0 | **0.184** | 0.0002 |
| **Cluster covariates** | | | |
| **Aedes density_c_ (AI_c_)** |  |  | <0.0001^b^ |
|  | 0 | Reference |  |
|  | 0-1 | **0.056** | <0.0001 |
|  | 1.1-3 | **0.036** | <0.0001 |
|  | >3 | **0.045** | <0.0001 |
| **Aedes density indoors_c_ (AI_c__in)** |  |  | <0.0001^b^ |
|  | 0 | Reference |  |
|  | 0-0.65 | **0.030** | <0.0001 |
|  | 0.66-1 | **0.020** | 0.016 |
|  | >1 | **0.038** | <0.0001 |
| **DENV infected Aedes**  **(AI_c_ DENV+)** |  |  | <0.0001^b^ |
|  | 0 | Reference |  |
|  | >0 | **0.000** | <0.0001 |
| **Breteau Index (BI)** |  |  | <0.0001^b^ |
|  | 0 | Reference |  |
|  | 0-34 | **-0.074** | <0.0001 |
|  | 34-68 | **-0.203** | <0.0001 |
|  | >68 | -0.030 | 0.0905 |
| **Container Index_c_ (CI_c_)** |  |  | <0.0001^b^ |
|  | 0 | Reference |  |
|  | 0-10 | **0.115** | <0.0001 |
|  | 10-20 | **0.063** | <0.0001 |
|  | >20 | **0.206** | <0.0001 |
| **House Index (HI)** |  |  | <0.0001^b^ |
|  | 0 | Reference |  |
|  | 0-33 | **-0.148** | <0.0001 |
|  | 33-65 | **0.586** | <0.0001 |
|  | >65 | **0.468** | <0.0001 |
| **Pupae per House (PHI_c_)** |  |  | <0.0001^b^ |
|  | 0 | Reference |  |
|  | 0-1 | **0.467** | <0.0001 |
|  | 1-2.9 | -0.002 | 0.7809 |
|  | >2.9 | **-0.062** | <0.0001 |
| **Pupae per Person Index (PPI_c_)** |  |  | <0.0001^b^ |
|  | 0 | Reference |  |
|  | 0-0.25 | **-0.062** | 0.0001 |
|  | 0.25-0.75 | **0.422** | <0.0001 |
|  | >0.75 | **0.202** | <0.0001 |
| **Province covariates** | | | |
| **Season** |  |  | <0.0001^b^ |
|  | Cool | Reference |  |
|  | Hot | **0.132** | <0.0001 |
|  | Rainy | **0.108** | <0.0001 |
| **Vector control intervention** |  |  | 0.8091^b^ |
|  | Control | Reference |  |
|  | Pyriproxyfen | -0.001 | 0.8091 |
| **Temperature maximum (°C)** |  | **-0.006** | <0.0001^b^ |
| **Temperature minimum (°C)** |  | **-0.003** | <0.0001^b^ |
| **Relative Humidity (%)** |  | **0.002** | <0.0001^b^ |
| **Rainfall (mm)** |  | **0.0002** | <0.0001^b^ |
| ^a^ Difference between each class and the reference class | | | |
| ^b^ Likelihood ratio test to assess the global effect of variable. | | | |
